# Miscellany

**Published:** 1905-08

**Authors:** 


### Miscellany.

French Dental College in Canada.—L'École de Chirurgie Dentaire de l'Université de Laval, at Montreal (Canada), is the only dental college in America in which the instruction is given entirely in the French language. Under another name and built upon a different basis, it has taken the place of the French faculty of the Dental College of Quebec. The courses were opened with an increased number of students. The professors are prominent dentists of high standing. They are Drs. Eudore Dubeau, M. Josef Nolin, M. Y. G. A. Gendreau. We express our best wishes for the complete success of their school.—*Le Laboratoire*, Paris.—C. M. L.

---

Teeth Receding in Vulcanization.—"How is it," asks a dentist, "that the teeth often recede from the caoutchouc, leaving an empty space? I have employed different kinds of rubber and bent the pins, yet experience frequently the same inconvenience."

*Answer:* Follow closely the instructions explained in every box of the rubber.

Let the temperature of the vulcanizer go up very slowly and vulcanize rather under than above the normal degree. Take more time for the whole process.

Heat your flask in boiling water, and your rubber over steam, taking care that it does not burn. (C. Soulard, Lyon, *Le Laboratoire.*)—C. M. L.

A New Property of Aluminum.—" A German investigator," says the *Scientific American,* " has discovered an exceedingly valuable and important property of aluminum, which consists in its application as a whetting agent, the effect produced on cutlery set with it being most astonishing. Though a metal, aluminum possesses the structure of a fine stone, has a strong dissolving power, and develops, upon use for honing, an exceedingly fine metal-setting substance of greasy feel, while showing great adhesion to steel. The knives, etc., treated with it quickly obtain such a fine, razor-like edge that even the best whetstone cannot produce a like result. Thus, knives which had been carefully set on a whetstone, when magnified a thousand times, still exhibited irregularities and roughness on the edge, while the edge of a knife sharpened on aluminum, upon exactly the same magnification, appeared as a straight, smooth line."—*British Dental Journal.*

---

Dr. Bartler recommends a saturated solution of shellac in borax water for the coating of plaster casts. It combines well with the plaster without forming a layer (schicht) upon it. This method facilitates also the separating of the cast from the impression.—(*Asch's Wiener Fachblatt.*)

---

If the flask is placed in the vulcanizer so that it stands above the water, a much stronger plate will be the result, as the rubber hardens best in steam. (*Asch's Quarterly Circular.*)—C. M. L.

---

To prevent the bubbling of the borax during the soldering process, mix a minimum of gum-arabic and water with the borax on a glass slab.

---

Before closing the flask use a piece of rubber dam coated with soap instead of the muslin. It will give an excellent result. (*Asch's Quarterly.*)

An "Antivolat" measure for somnoform, ethyl chloride, or any other liquid anæsthetic, is a double glass graduate with a closed space between the inside and outside to serve as a non-conductor of heat, and provided with a cover. It is extremely difficult to measure accurately very volatile liquids with the ordinary graduate. The heat of the vessel and of the surrounding atmosphere causes a serious loss by rapid evaporation. With this measure, the small portion first poured in, by its rapid evaporation, chills the vessel, reducing its temperature below the boiling-point of the volatile liquid and preventing any further loss. The inner chamber, being insulated, so retards evaporation that if filled a portion will remain after twelve hours' time.

